# *cis*-Bis[1-allyl-3-(2-pyrid­yl-κ*N*)thio­ureato-κ*S*]palladium(II)

**DOI:** 10.1107/S1600536809029262

**Published:** 2009-08-12

**Authors:** Svitlana Orysyk, Volodimir Bon, Vasily Pekhnyo

**Affiliations:** a Institute of General and Inorganic Chemistry, NAS Ukraine, Kyiv, Prosp. Palladina 32/34, 03680 Ukraine

## Abstract

Yellow plate-like shaped crystals of the title compound, [Pd(C_9_H_10_N_3_S)_2_], were obtained by ligand-exchange reaction between palladium(II) acetyl­acetonate and the corresponding organic reagent at room temperature. The Pd^II^ atom shows a slightly distorted square-planar coordination geometry consisting of two ligand mol­ecules in a *cis* conformation that bind in their thio­lic tautomeric form. Weak inter­molecular Pd⋯H inter­actions with Pd—H distances of 3.328 (2) Å were observed in the crystal structure. The three-dimensional network of the crystal structure is realized by weak inter­molecular C—H⋯N, N—H⋯N and C—H⋯S hydrogen bonds.

## Related literature

For a related structure, see: Bon *et al.* (2007 ▶). For the anti­tumoral properties of Pd compounds, see: Upadhayaya *et al.* (2009 ▶), Hernández *et al.* (2008 ▶).
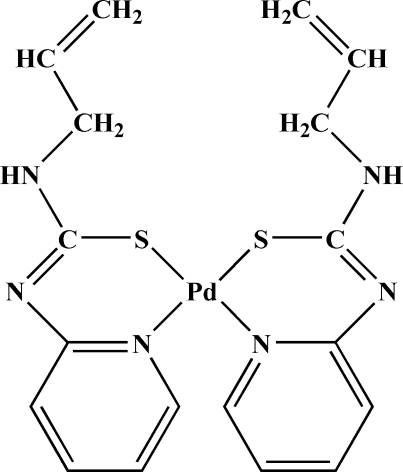


## Experimental

### 

#### Crystal data

[Pd(C_9_H_10_N_3_S)_2_]
*M* *_r_* = 490.96
Monoclinic, 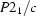

*a* = 10.8976 (6) Å
*b* = 8.9730 (5) Å
*c* = 21.798 (1) Å
β = 113.624 (2)°
*V* = 1952.9 (2) Å^3^
*Z* = 4
Mo *K*α radiation
μ = 1.18 mm^−1^
*T* = 173 K
0.51 × 0.21 × 0.05 mm

#### Data collection

Bruker APEXII CCD diffractometer
Absorption correction: numerical (*SADABS*; Bruker, 2005 ▶) *T*_min_ = 0.583, *T*_max_ = 0.941
17417 measured reflections
4010 independent reflections
3522 reflections with *I* > 2σ(*I*)
*R*_int_ = 0.026

#### Refinement

*R*[*F* ^2^ > 2σ(*F* ^2^)] = 0.022
*wR*(*F* ^2^) = 0.055
*S* = 1.03
4010 reflections
272 parameters
3 restraints
H atoms treated by a mixture of independent and constrained refinement
Δρ_max_ = 0.58 e Å^−3^
Δρ_min_ = −0.37 e Å^−3^

### 

Data collection: *APEX2* (Bruker, 2005 ▶); cell refinement: *SAINT* (Bruker, 2005 ▶); data reduction: *SAINT*; program(s) used to solve structure: *SHELXS97* (Sheldrick, 2008 ▶); program(s) used to refine structure: *SHELXL97* (Sheldrick, 2008 ▶); molecular graphics: *SHELXTL* (Sheldrick, 2008 ▶); software used to prepare material for publication: *publCIF* (Westrip, 2009 ▶).

## Supplementary Material

Crystal structure: contains datablocks I, global. DOI: 10.1107/S1600536809029262/im2129sup1.cif

Structure factors: contains datablocks I. DOI: 10.1107/S1600536809029262/im2129Isup2.hkl

Additional supplementary materials:  crystallographic information; 3D view; checkCIF report

## Figures and Tables

**Table 1:** Hydrogen-bond geometry (Å, °)

*D*—H⋯*A*	*D*—H	H⋯*A*	*D*⋯*A*	*D*—H⋯*A*
C3—H3*A*⋯N2^i^	0.95	2.64	3.511 (3)	152
C14—H14*A*⋯N5^ii^	0.95	2.57	3.376 (3)	143
N3—H3*N*⋯N3^iii^	0.78 (3)	2.84 (3)	3.425 (4)	133 (2)
C4—H4*A*⋯S2^iv^	0.95	2.98	3.728 (2)	136
C12—H12*A*⋯S1^v^	0.95	3.02	3.910 (2)	156
C18*B*—H18*D*⋯S1^vi^	0.95	2.95	3.85 (1)	160
C7—H7*A*⋯S2^iii^	0.99	2.85	3.825 (3)	167

Symmetry codes: (i) 
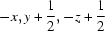
; (ii) 
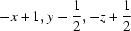
; (iii) 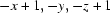; (iv) 
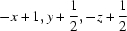
; (v) 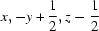; (vi) 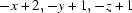.

**Fig. 1.**
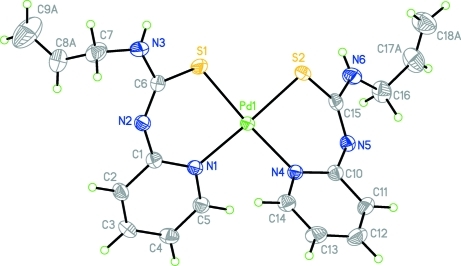
The title compound showing 50% probability displacement ellipsoids for the non-hydrogen atoms. Only one position is shown for disordered allyl fragments

**Fig. 2.**
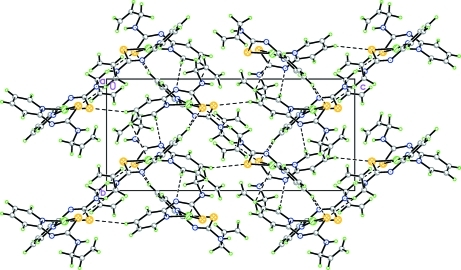
Crystal packing of title compound, projection down the a axis. Dashed lines indicate hydrogen bonds.

## supplementary crystallographic information

### Comment

Palladium complexes with carbothioamide derivatives as organic ligands attract ongoing scientific interest due to their cytotoxic, antitumoral, antifungal and antimicrobial activities (Upadhayaya *et al.* 2009; Hernández *et al.* 2008)

The asymmetric unit of the title compound contains one molecule of the complex (Fig. 1). Palladium shows a slightly distorted square-planar coordination geometry with a mean deviation from the plane of 0.0679 (2) Å. Two molecules of the organic ligand chelate the palladium in *cis*-conformation *via* carbothioamide sulfur and pyridine nitrogen atoms. The observed bond lengths of the carbothioamide fragment [C(6)—S(1) 1.752 (2), C(15)—S(2) 1.753 (2), C(6)—N(2) 1.301 (3) and C(15)—N(5) 1.302 (3) Å] indicate the thiolic tautomeric form of the thiourea derivative. The six-membered metallacycle exhibits a non-planar geometry with torsion angles Pd(1)—S(1)—C(6)—N(2) 142.8 (2) and Pd(1)—S(2)—C(15)—N(5) 134.1 (2)°. The allyl substituents of both coordinated ligand molecules are disordered over two positions with occupancies of 0.7 and 0.3, respectively. The crystal structure of the title compound shows weak intermolecular Pd···H interactions with d(Pd—H) = 3.328 Å. The 3-D network is realized by weak C—H···N, N—H···N and C—H···S intermolecular hydrogen bonds (Fig.2, Table 1).

### Experimental

Single crystals of title compound were synthesized by a ligand exchange reaction between 5 ml (5x10^-3 ^M) of a solution of palladium(II) acetylacetonate in chloroform and 5 ml (10^-2^*M*) of an ethanolic solution of the organic ligand. After staying one month in a dark place the layered mixture becomes homogeneous and yellow plate-like shaped crystals were grown.

### Refinement

Disorder of both allyl fragments has been observed in the molecular structure of the title compound. Disordered allyl substituents C8, C9 and C17, C18 were treated with occupancies 0.71/0.29 and 0.70/0.30, respectively, and refined with equal ADP for both parts. H atoms bonded to N were located in a difference map and refined freely. Other H atoms were positioned geometrically and refined using a riding model with C—H = 0.99 Å for CH_2_ [*U*_iso_(H) = 1.2Ueq(C)] and C—H = 0.95 Å for CH [*U*_iso_(H) = 1.2Ueq(C)].

### Crystal data

**Table:**

[Pd(C_9_H_10_N_3_S)_2_]	*F*(000) = 992
*M**_r_* = 490.96	*D*_x_ = 1.670 Mg m^−^^3^
Monoclinic, *P*2_1_/*c*	Melting point: 343 K
Hall symbol: -P 2ybc	Mo *K*α radiation, λ = 0.71073 Å
*a* = 10.8976 (6) Å	Cell parameters from 8451 reflections
*b* = 8.9730 (5) Å	θ = 3.0–26.4°
*c* = 21.798 (1) Å	µ = 1.18 mm^−^^1^
β = 113.624 (2)°	*T* = 173 K
*V* = 1952.9 (2) Å^3^	Plate, yellow
*Z* = 4	0.51 × 0.21 × 0.05 mm

### Data collection

**Table:**

Bruker APEXII CCD diffractometer	4010 independent reflections
Radiation source: fine-focus sealed tube	3522 reflections with *I* > 2σ(*I*)
graphite	*R*_int_ = 0.026
Detector resolution: 8.26 pixels mm^-1^	θ_max_ = 26.4°, θ_min_ = 2.0°
φ and ω scans	*h* = −13→13
Absorption correction: numerical (*SADABS*; Bruker, 2005)	*k* = −11→11
*T*_min_ = 0.583, *T*_max_ = 0.941	*l* = −26→27
17417 measured reflections	

### Refinement

**Table:**

Refinement on *F*^2^	Primary atom site location: structure-invariant direct methods
Least-squares matrix: full	Secondary atom site location: difference Fourier map
*R*[*F*^2^ > 2σ(*F*^2^)] = 0.022	Hydrogen site location: inferred from neighbouring sites
*wR*(*F*^2^) = 0.055	H atoms treated by a mixture of independent and constrained refinement
*S* = 1.03	*w* = 1/[σ^2^(*F*_o_^2^) + (0.02*P*)^2^ + 1.8761*P*] where *P* = (*F*_o_^2^ + 2*F*_c_^2^)/3
4010 reflections	(Δ/σ)_max_ = 0.001
272 parameters	Δρ_max_ = 0.58 e Å^−^^3^
3 restraints	Δρ_min_ = −0.37 e Å^−^^3^

### Special details

**Table:**

Geometry. All e.s.d.'s (except the e.s.d. in the dihedral angle between two l.s. planes) are estimated using the full covariance matrix. The cell e.s.d.'s are taken into account individually in the estimation of e.s.d.'s in distances, angles and torsion angles; correlations between e.s.d.'s in cell parameters are only used when they are defined by crystal symmetry. An approximate (isotropic) treatment of cell e.s.d.'s is used for estimating e.s.d.'s involving l.s. planes.
Refinement. Refinement of *F*^2^ against ALL reflections. The weighted *R*-factor *wR* and goodness of fit *S* are based on *F*^2^, conventional *R*-factors *R* are based on *F*, with *F* set to zero for negative *F*^2^. The threshold expression of *F*^2^ > σ(*F*^2^) is used only for calculating *R*-factors(gt) *etc*. and is not relevant to the choice of reflections for refinement. *R*-factors based on *F*^2^ are statistically about twice as large as those based on *F*, and *R*- factors based on ALL data will be even larger.

### Fractional atomic coordinates and isotropic or equivalent isotropic displacement parameters (Å^2^)

**Table:**

	*x*	*y*	*z*	*U*_iso_*/*U*_eq_	Occ. (<1)
Pd1	0.504182 (15)	0.273165 (18)	0.331038 (7)	0.02144 (6)	
S1	0.48920 (6)	0.26120 (8)	0.43136 (3)	0.03554 (15)	
S2	0.72854 (5)	0.24180 (7)	0.38621 (3)	0.02877 (13)	
N1	0.30161 (17)	0.3217 (2)	0.28592 (8)	0.0226 (4)	
N2	0.22927 (17)	0.1688 (2)	0.35666 (9)	0.0246 (4)	
N3	0.3409 (2)	0.0682 (3)	0.45961 (10)	0.0356 (5)	
N4	0.52967 (17)	0.26693 (19)	0.24180 (8)	0.0216 (4)	
N5	0.73705 (17)	0.4037 (2)	0.27993 (8)	0.0238 (4)	
N6	0.88430 (19)	0.4612 (2)	0.38499 (10)	0.0330 (5)	
C1	0.2066 (2)	0.2704 (2)	0.30613 (10)	0.0231 (4)	
C2	0.0738 (2)	0.3203 (3)	0.27361 (11)	0.0282 (5)	
H2A	0.0067	0.2803	0.2862	0.034*	
C3	0.0396 (2)	0.4252 (3)	0.22420 (11)	0.0303 (5)	
H3A	−0.0500	0.4600	0.2033	0.036*	
C4	0.1382 (2)	0.4802 (3)	0.20512 (11)	0.0299 (5)	
H4A	0.1177	0.5536	0.1710	0.036*	
C5	0.2656 (2)	0.4259 (2)	0.23664 (11)	0.0268 (5)	
H5A	0.3327	0.4633	0.2233	0.032*	
C6	0.3393 (2)	0.1622 (3)	0.41056 (10)	0.0262 (5)	
C7	0.2192 (3)	0.0080 (3)	0.46317 (13)	0.0396 (6)	
H7A	0.2445	−0.0645	0.5005	0.048*	
H7B	0.1673	−0.0461	0.4211	0.048*	
C8A	0.1310 (4)	0.1276 (5)	0.4737 (2)	0.0501 (10)	0.711 (5)
H8AA	0.0885	0.1963	0.4383	0.060*	0.711 (5)
C9A	0.1099 (12)	0.1423 (10)	0.5274 (5)	0.0819 (19)	0.711 (5)
H9AA	0.1507	0.0755	0.5638	0.098*	0.711 (5)
H9AB	0.0534	0.2198	0.5307	0.098*	0.711 (5)
C8B	0.1993 (11)	0.0863 (13)	0.5186 (6)	0.0501 (10)	0.289 (5)
H8BA	0.2630	0.0696	0.5630	0.060*	0.289 (5)
C9B	0.103 (3)	0.173 (3)	0.5105 (16)	0.0819 (19)	0.289 (5)
H9BA	0.0377	0.1924	0.4668	0.098*	0.289 (5)
H9BB	0.0962	0.2199	0.5481	0.098*	0.289 (5)
C10	0.6369 (2)	0.3219 (2)	0.23255 (10)	0.0230 (4)	
C11	0.6465 (2)	0.3015 (3)	0.17078 (11)	0.0332 (5)	
H11A	0.7189	0.3452	0.1635	0.040*	
C12	0.5531 (3)	0.2197 (3)	0.12094 (12)	0.0377 (6)	
H12A	0.5613	0.2041	0.0797	0.045*	
C13	0.4457 (2)	0.1597 (3)	0.13180 (11)	0.0350 (5)	
H13A	0.3798	0.1013	0.0984	0.042*	
C14	0.4373 (2)	0.1868 (3)	0.19154 (11)	0.0289 (5)	
H14A	0.3628	0.1476	0.1984	0.035*	
C15	0.7807 (2)	0.3775 (2)	0.34392 (10)	0.0240 (4)	
C16	0.9495 (2)	0.5766 (3)	0.36190 (13)	0.0380 (6)	
H16A	0.9016	0.6715	0.3598	0.046*	
H16B	0.9375	0.5515	0.3156	0.046*	
C17A	1.0931 (6)	0.6027 (8)	0.4014 (4)	0.0486 (15)	0.700 (9)
H17A	1.1296	0.6914	0.3918	0.058*	0.700 (9)
C18A	1.1731 (5)	0.5195 (7)	0.4466 (3)	0.0540 (13)	0.700 (9)
H18A	1.1419	0.4294	0.4581	0.065*	0.700 (9)
H18B	1.2645	0.5473	0.4691	0.065*	0.700 (9)
C17B	1.0811 (14)	0.6032 (17)	0.4263 (6)	0.037 (3)	0.300 (9)
H17B	1.0745	0.6469	0.4645	0.044*	0.300 (9)
C18B	1.2000 (13)	0.5671 (16)	0.4290 (7)	0.0540 (13)	0.300 (9)
H18C	1.2085	0.5233	0.3912	0.065*	0.300 (9)
H18D	1.2775	0.5847	0.4687	0.065*	0.300 (9)
H6N	0.904 (2)	0.452 (3)	0.4237 (13)	0.027 (7)*	
H3N	0.404 (3)	0.072 (3)	0.4936 (13)	0.038 (8)*	

### Atomic displacement parameters (Å^2^)

**Table:**

	*U*^11^	*U*^22^	*U*^33^	*U*^12^	*U*^13^	*U*^23^
Pd1	0.01747 (9)	0.02785 (10)	0.01790 (9)	−0.00036 (7)	0.00594 (6)	0.00161 (6)
S1	0.0235 (3)	0.0626 (4)	0.0191 (3)	−0.0085 (3)	0.0070 (2)	−0.0001 (3)
S2	0.0197 (3)	0.0401 (3)	0.0239 (3)	0.0029 (2)	0.0061 (2)	0.0081 (2)
N1	0.0208 (9)	0.0246 (9)	0.0209 (9)	0.0000 (7)	0.0067 (7)	0.0016 (7)
N2	0.0226 (9)	0.0260 (9)	0.0244 (9)	−0.0010 (8)	0.0086 (7)	0.0030 (7)
N3	0.0307 (11)	0.0493 (13)	0.0246 (10)	0.0025 (10)	0.0089 (9)	0.0118 (9)
N4	0.0207 (9)	0.0228 (9)	0.0203 (9)	−0.0008 (7)	0.0071 (7)	0.0007 (7)
N5	0.0204 (9)	0.0259 (10)	0.0247 (9)	−0.0012 (7)	0.0084 (7)	0.0003 (7)
N6	0.0257 (11)	0.0419 (12)	0.0250 (11)	−0.0068 (9)	0.0035 (9)	−0.0041 (9)
C1	0.0230 (11)	0.0212 (10)	0.0240 (10)	−0.0029 (9)	0.0082 (9)	−0.0029 (8)
C2	0.0198 (11)	0.0314 (12)	0.0317 (12)	−0.0025 (9)	0.0086 (9)	0.0007 (9)
C3	0.0205 (11)	0.0319 (13)	0.0320 (12)	0.0035 (9)	0.0038 (9)	0.0020 (10)
C4	0.0302 (12)	0.0273 (12)	0.0272 (11)	0.0008 (10)	0.0064 (9)	0.0053 (9)
C5	0.0253 (11)	0.0292 (12)	0.0253 (11)	−0.0022 (9)	0.0093 (9)	0.0022 (9)
C6	0.0241 (11)	0.0323 (12)	0.0244 (11)	0.0032 (9)	0.0120 (9)	0.0011 (9)
C7	0.0474 (16)	0.0395 (14)	0.0343 (13)	−0.0108 (12)	0.0188 (12)	0.0048 (11)
C8A	0.042 (2)	0.068 (3)	0.049 (2)	0.0008 (19)	0.0275 (18)	0.005 (2)
C9A	0.133 (4)	0.060 (5)	0.081 (7)	0.006 (4)	0.072 (5)	0.018 (3)
C8B	0.042 (2)	0.068 (3)	0.049 (2)	0.0008 (19)	0.0275 (18)	0.005 (2)
C9B	0.133 (4)	0.060 (5)	0.081 (7)	0.006 (4)	0.072 (5)	0.018 (3)
C10	0.0230 (11)	0.0209 (10)	0.0247 (10)	0.0013 (8)	0.0091 (9)	0.0024 (8)
C11	0.0315 (13)	0.0446 (15)	0.0269 (12)	−0.0040 (11)	0.0153 (10)	0.0024 (10)
C12	0.0433 (15)	0.0479 (15)	0.0233 (12)	−0.0028 (12)	0.0148 (11)	−0.0041 (10)
C13	0.0388 (14)	0.0365 (14)	0.0238 (11)	−0.0081 (11)	0.0064 (10)	−0.0074 (10)
C14	0.0263 (12)	0.0299 (12)	0.0275 (11)	−0.0055 (10)	0.0077 (9)	−0.0019 (9)
C15	0.0191 (10)	0.0245 (11)	0.0281 (11)	0.0034 (9)	0.0094 (9)	−0.0023 (9)
C16	0.0321 (13)	0.0382 (14)	0.0427 (14)	−0.0109 (11)	0.0139 (11)	−0.0101 (11)
C17A	0.033 (3)	0.045 (3)	0.063 (4)	−0.013 (2)	0.014 (3)	−0.002 (3)
C18A	0.038 (3)	0.059 (4)	0.054 (3)	−0.009 (2)	0.0074 (19)	0.005 (2)
C17B	0.039 (6)	0.035 (5)	0.045 (7)	−0.015 (4)	0.026 (6)	−0.011 (5)
C18B	0.038 (3)	0.059 (4)	0.054 (3)	−0.009 (2)	0.0074 (19)	0.005 (2)

### Geometric parameters (Å, °)

**Table:**

Pd1—N1	2.0713 (17)	C7—H7A	0.9900
Pd1—N4	2.0730 (17)	C7—H7B	0.9900
Pd1—S1	2.2598 (6)	C8A—C9A	1.288 (7)
Pd1—S2	2.2686 (6)	C8A—H8AA	0.9500
S1—C6	1.752 (2)	C9A—H9AA	0.9500
S2—C15	1.753 (2)	C9A—H9AB	0.9500
N1—C1	1.358 (3)	C8B—C9B	1.261 (17)
N1—C5	1.358 (3)	C8B—H8BA	0.9500
N2—C6	1.301 (3)	C9B—H9BA	0.9500
N2—C1	1.374 (3)	C9B—H9BB	0.9500
N3—C6	1.357 (3)	C10—C11	1.404 (3)
N3—C7	1.463 (3)	C11—C12	1.367 (3)
N3—H3N	0.78 (3)	C11—H11A	0.9500
N4—C10	1.355 (3)	C12—C13	1.392 (4)
N4—C14	1.359 (3)	C12—H12A	0.9500
N5—C15	1.302 (3)	C13—C14	1.363 (3)
N5—C10	1.376 (3)	C13—H13A	0.9500
N6—C15	1.353 (3)	C14—H14A	0.9500
N6—C16	1.455 (3)	C16—C17A	1.471 (7)
N6—H6N	0.79 (2)	C16—C17B	1.572 (13)
C1—C2	1.405 (3)	C16—H16A	0.9900
C2—C3	1.365 (3)	C16—H16B	0.9900
C2—H2A	0.9500	C17A—C18A	1.265 (9)
C3—C4	1.389 (3)	C17A—H17A	0.9500
C3—H3A	0.9500	C18A—H18A	0.9500
C4—C5	1.368 (3)	C18A—H18B	0.9500
C4—H4A	0.9500	C17B—C18B	1.31 (2)
C5—H5A	0.9500	C17B—H17B	0.9500
C7—C8B	1.487 (11)	C18B—H18C	0.9500
C7—C8A	1.519 (5)	C18B—H18D	0.9500
			
N1—Pd1—N4	94.48 (7)	C9A—C8A—H8AA	117.8
N1—Pd1—S1	89.59 (5)	C7—C8A—H8AA	117.8
N4—Pd1—S1	174.63 (5)	C8A—C9A—H9AA	120.0
N1—Pd1—S2	174.24 (5)	C8A—C9A—H9AB	120.0
N4—Pd1—S2	88.44 (5)	H9AA—C9A—H9AB	120.0
S1—Pd1—S2	87.80 (2)	C9B—C8B—C7	124.2 (18)
C6—S1—Pd1	101.25 (7)	C9B—C8B—H8BA	117.9
C15—S2—Pd1	98.20 (7)	C7—C8B—H8BA	117.9
C1—N1—C5	118.27 (18)	C8B—C9B—H9BA	120.0
C1—N1—Pd1	125.52 (14)	C8B—C9B—H9BB	120.0
C5—N1—Pd1	115.82 (14)	H9BA—C9B—H9BB	120.0
C6—N2—C1	123.98 (19)	N4—C10—N5	123.65 (18)
C6—N3—C7	123.1 (2)	N4—C10—C11	119.71 (19)
C6—N3—H3N	116 (2)	N5—C10—C11	116.58 (19)
C7—N3—H3N	116 (2)	C12—C11—C10	120.9 (2)
C10—N4—C14	118.47 (18)	C12—C11—H11A	119.5
C10—N4—Pd1	125.27 (14)	C10—C11—H11A	119.5
C14—N4—Pd1	115.87 (14)	C11—C12—C13	118.8 (2)
C15—N5—C10	123.16 (18)	C11—C12—H12A	120.6
C15—N6—C16	124.1 (2)	C13—C12—H12A	120.6
C15—N6—H6N	116.6 (18)	C14—C13—C12	118.5 (2)
C16—N6—H6N	118.9 (18)	C14—C13—H13A	120.8
N1—C1—N2	124.70 (19)	C12—C13—H13A	120.8
N1—C1—C2	119.55 (19)	N4—C14—C13	123.6 (2)
N2—C1—C2	115.73 (19)	N4—C14—H14A	118.2
C3—C2—C1	121.2 (2)	C13—C14—H14A	118.2
C3—C2—H2A	119.4	N5—C15—N6	117.3 (2)
C1—C2—H2A	119.4	N5—C15—S2	129.22 (17)
C2—C3—C4	118.8 (2)	N6—C15—S2	113.46 (16)
C2—C3—H3A	120.6	N6—C16—C17A	117.6 (3)
C4—C3—H3A	120.6	N6—C16—C17B	101.3 (5)
C5—C4—C3	118.3 (2)	N6—C16—H16A	107.9
C5—C4—H4A	120.8	C17A—C16—H16A	107.9
C3—C4—H4A	120.8	C17B—C16—H16A	100.7
N1—C5—C4	123.8 (2)	N6—C16—H16B	107.9
N1—C5—H5A	118.1	C17A—C16—H16B	107.9
C4—C5—H5A	118.1	C17B—C16—H16B	130.2
N2—C6—N3	117.1 (2)	H16A—C16—H16B	107.2
N2—C6—S1	129.58 (17)	C18A—C17A—C16	127.0 (6)
N3—C6—S1	113.29 (17)	C18A—C17A—H17A	116.5
N3—C7—C8B	107.4 (4)	C16—C17A—H17A	116.5
N3—C7—C8A	112.9 (2)	C17A—C18A—H18A	120.0
N3—C7—H7A	109.0	C17A—C18A—H18B	120.0
C8B—C7—H7A	74.1	H18A—C18A—H18B	120.0
C8A—C7—H7A	109.0	C18B—C17B—C16	122.2 (12)
N3—C7—H7B	109.0	C18B—C17B—H17B	118.9
C8B—C7—H7B	140.3	C16—C17B—H17B	118.9
C8A—C7—H7B	109.0	C17B—C18B—H18C	120.0
H7A—C7—H7B	107.8	C17B—C18B—H18D	120.0
C9A—C8A—C7	124.3 (5)	H18C—C18B—H18D	120.0
			
N1—Pd1—S1—C6	42.71 (9)	C6—N3—C7—C8B	−106.2 (5)
S2—Pd1—S1—C6	−142.43 (8)	C6—N3—C7—C8A	−63.7 (3)
N4—Pd1—S2—C15	47.84 (9)	N3—C7—C8A—C9A	−112.2 (8)
S1—Pd1—S2—C15	−136.00 (7)	C8B—C7—C8A—C9A	−22.0 (10)
N4—Pd1—N1—C1	143.98 (17)	N3—C7—C8B—C9B	113.5 (19)
S1—Pd1—N1—C1	−32.53 (17)	C8A—C7—C8B—C9B	8.4 (18)
N4—Pd1—N1—C5	−43.35 (16)	C14—N4—C10—N5	−179.6 (2)
S1—Pd1—N1—C5	140.15 (15)	Pd1—N4—C10—N5	−7.2 (3)
N1—Pd1—N4—C10	140.26 (17)	C14—N4—C10—C11	3.2 (3)
S2—Pd1—N4—C10	−34.78 (16)	Pd1—N4—C10—C11	175.61 (16)
N1—Pd1—N4—C14	−47.14 (16)	C15—N5—C10—N4	36.3 (3)
S2—Pd1—N4—C14	137.82 (15)	C15—N5—C10—C11	−146.4 (2)
C5—N1—C1—N2	−177.9 (2)	N4—C10—C11—C12	−3.8 (4)
Pd1—N1—C1—N2	−5.4 (3)	N5—C10—C11—C12	178.9 (2)
C5—N1—C1—C2	3.2 (3)	C10—C11—C12—C13	1.7 (4)
Pd1—N1—C1—C2	175.69 (15)	C11—C12—C13—C14	0.8 (4)
C6—N2—C1—N1	35.3 (3)	C10—N4—C14—C13	−0.7 (3)
C6—N2—C1—C2	−145.8 (2)	Pd1—N4—C14—C13	−173.80 (19)
N1—C1—C2—C3	−3.3 (3)	C12—C13—C14—N4	−1.4 (4)
N2—C1—C2—C3	177.6 (2)	C10—N5—C15—N6	176.7 (2)
C1—C2—C3—C4	1.5 (3)	C10—N5—C15—S2	−0.6 (3)
C2—C3—C4—C5	0.3 (3)	C16—N6—C15—N5	−0.5 (3)
C1—N1—C5—C4	−1.4 (3)	C16—N6—C15—S2	177.21 (18)
Pd1—N1—C5—C4	−174.65 (18)	Pd1—S2—C15—N5	−45.9 (2)
C3—C4—C5—N1	−0.4 (4)	Pd1—S2—C15—N6	136.77 (15)
C1—N2—C6—N3	172.1 (2)	C15—N6—C16—C17A	−148.5 (4)
C1—N2—C6—S1	−6.3 (3)	C15—N6—C16—C17B	−165.4 (6)
C7—N3—C6—N2	−17.4 (3)	N6—C16—C17A—C18A	13.5 (10)
C7—N3—C6—S1	161.29 (19)	C17B—C16—C17A—C18A	61.2 (18)
Pd1—S1—C6—N2	−37.2 (2)	N6—C16—C17B—C18B	112.2 (14)
Pd1—S1—C6—N3	144.37 (16)	C17A—C16—C17B—C18B	−25.9 (13)

### Hydrogen-bond geometry (Å, °)

**Table:**

*D*—H···*A*	*D*—H	H···*A*	*D*···*A*	*D*—H···*A*
C3—H3A···N2^i^	0.95	2.64	3.511 (3)	152
C14—H14A···N5^ii^	0.95	2.57	3.376 (3)	143
N3—H3N···N3^iii^	0.78 (3)	2.84 (3)	3.425 (4)	133 (2)
C4—H4A···S2^iv^	0.95	2.98	3.728 (2)	136
C12—H12A···S1^v^	0.95	3.02	3.910 (2)	156
C18B—H18D···S1^vi^	0.95	2.95	3.85 (1)	160
C7—H7A···S2^iii^	0.99	2.85	3.825 (3)	167

Symmetry codes: (i) −*x*, *y*+1/2, −*z*+1/2; (ii) −*x*+1, *y*−1/2, −*z*+1/2; (iii) −*x*+1, −*y*, −*z*+1; (iv) −*x*+1, *y*+1/2, −*z*+1/2; (v) *x*, −*y*+1/2, *z*−1/2; (vi) −*x*+2, −*y*+1, −*z*+1.
